# Identification of heat-tolerant mungbean genotypes through morpho-physiological evaluation and key gene expression analysis

**DOI:** 10.3389/fgene.2024.1482956

**Published:** 2024-10-10

**Authors:** Ragini Bhardwaj, Gayacharan, Bharat H. Gawade, Pooja Pathania, Akshay Talukdar, Prakash Kumar, Suphiya Khan, Gyanendra Pratap Singh

**Affiliations:** ^1^ ICAR-National Bureau of Plant Genetic Resources, New Delhi, India; ^2^ Department of Bioscience and Biotechnology, Banasthali Vidyapith University, Vanasthali, India; ^3^ Division of Genetics, ICAR-Indian Agricultural Research Institute, New Delhi, India; ^4^ Division of Statistical Genetics, ICAR-Indian Agricultural Statistics Research Institute, New Delhi, India

**Keywords:** green gram, abiotic stress tolerance, physiological parameters, gene expression, climate resilience

## Abstract

Mungbean plays a significant role in global food and nutritional security. However, the recent drastic rise in atmospheric temperature has posed an imminent threat to mungbean cultivation. Therefore, this study investigates the growth and physiological changes of 87 mungbean germplasm lines under heat stress. Genotypes were examined using parameters including leaf area, chlorophyll content, membrane stability index (MSI), stomatal conductance, pollen viability, number of pods per cluster, number of pods per plant, number of seeds/pod, 100-seed weight and grain yield/plant under heat stress and control environments. A wide range of variation was observed for these traits among genotypes under heat stress and control environments. Genotypes were also identified with variable responses under both environments. The phenotypic expression of selected promising accessions was also validated in control environment conditions at the National Phytotron facility. The selected promising genotypes viz., IC76475, IC418452 and IC489062 validated their heat tolerance behavior for key candidate genes revealed by quantitative real-time PCR (qRT-PCR). These mungbean genotypes can act as potential resources in the mungbean improvement programs for heat stress tolerance. This study also provides a comprehensive understanding of the key mechanisms underlying heat tolerance in mungbean.

## 1 Introduction

Mungbean (*Vigna radiata* L. Wilczek) is a crucial summer season legume crop predominantly grown across tropical and subtropical regions of Asia. Its significance lies not only in its high nutritional value but also in its adaptability to diverse agro-climatic conditions. Globally, mungbean holds substantial agricultural importance with an annual production of approximately 6.0 million tons, which comes from an area of about 7.3 million hectares (Gayacharan et al., 2023). India stands out as the largest producer, contributing up to 41% of the global mungbean production, followed by Myanmar, Bangladesh and Pakistan (Schreinemachers et al., 2019). The short growing season of 65–90 days makes mungbean an ideal crop for multiple cropping systems prevalent in South and Southeast Asia (Lambrides and Godwin, 2007). Additionally, as a leguminous crop, mungbean plays a vital role in nitrogen fixation, thus enhancing soil fertility and breaking pest and disease cycles in cereal-based cropping patterns. Intercropping mungbean with cereals has been shown to significantly boost the yield of companion crops (Rachaputi et al., 2002). However, despite its immense significance, mungbean cultivation faces numerous challenges, particularly from environmental stressors such as drought, heat, and waterlogging. Among these, heat stress poses a significant threat to mungbean productivity, with temperatures exceeding 25°C at night adversely affecting pollen viability (Kumar et al., 2013). Although the mungbean plant is adapted to tropical and sub-tropical climatic conditions, prolonged atmospheric temperature rise above 40°C is very detrimental especially during flowering and pod development stages leading to substantial yield losses (Bhandari et al., 2016). The warmer temperature can also exacerbate pest and disease problems in mungbean crops. Insects such as the mung bean weevil (*Callosobruchus* spp.) and pathogens like mungbean yellow mosaic virus (MYMV) may become more prevalent. However, to a certain extent, mungbean production can be sustained by using the available mungbean variability in the genebank collections.

The crop genetic diversity is the key to sustainable crop improvement programs that allow researchers to develop new cultivars with improved yield, and tolerance to biotic and abiotic stresses. Mungbean genetic diversity primarily evolved in the Indian subcontinent as India is the center of its origin (Vavilov, 1926; Zukovskij, 1962). In the Indian sub-continent, the mungbean diversity is widely distributed from the foothills of the Himalayas to the Southern Peninsula and northeastern regions. The conservation efforts of the mungbean genetic diversity have led to *ex-situ* collections of over 43,000 mungbean accessions conserved worldwide (Gayacharan S. et al., 2020). As the mungbean has evolved in diverse climatic zones and food taste preferences, its rich genetic diversity is playing crucial roles in crop improvement programs. Genebank collections from ICAR-NBPGR, New Delhi, The World Vegetable Centre, Taiwan, and the U.S. Department of Agriculture (USDA), United States has played significant roles in crop improvement (Wang et al., 2018; Gayacharan et al., 2020). The *ex-situ* mungbean collections have been analyzed to understand the magnitude and nature of genetic diversity available in mungbean (Bisht et al., 1998; Schafleitner et al., 2015; Wang et al., 2018; Gayacharan et al., 2020), as well as utilized for identification sources for biotic and abiotic stress tolerance. Although limited studies have been done for the improvement of mungbean for heat stress tolerance, promising accessions for heat stress attributing traits are identified (Bhardwaj et al., 2023). Large-scale studies for germplasm evaluation against heat stress tolerance are still lacking.

Although, it is established across the plant species that in response to heat stress, plants deploy various defense mechanisms, unlike other major crops, mungbean lacks comprehensive molecular resources, such as identified QTLs, genes, and gene regulatory factors, related to heat stress tolerance. To date, only a few molecular studies have been conducted to elucidate the mechanisms underlying heat stress resilience in the crop. Notably, the upregulation of heat shock proteins (HSPs), antioxidant enzymes, osmoprotectants and signaling molecules plays a crucial role in mitigating the adverse effects of heat stress (Wang et al., 2004; Li et al., 2019). Transcriptomic studies have identified several heat-responsive genes in mungbean, including those encoding HSPs, late embryogenesis abundant (LEA) proteins, reactive oxygen species (ROS) enzymes and osmoprotectants such as proline and glycine-betaine (Li et al., 2015; Gupta et al., 2022). Albeit, advances in understanding the physiological and molecular responses of various crops to heat stress, there remains a gap in knowledge regarding genotype-specific responses in mungbean to elevated temperatures. Given the genetic diversity within mungbean germplasm, different genotypes may exhibit varying degrees of heat tolerance and employ distinct mechanisms to cope with heat stress. Therefore, comprehensive studies investigating the effect of heat stress on physiological parameters and related gene expression in different mungbean genotypes are essential for the identification of new sources of heat stress tolerance, which may further help in elucidating the underlying mechanisms of heat stress tolerance in mungbean.

In this context, the present study was aimed to investigate the effect of heat stress on morphological and physiological parameters in eighty-seven diverse mungbean genotypes. By employing a multifaceted approach integrating physiological, biochemical and molecular analyses, this study revealed the genotype-specific adaptive responses in response to heat stress in mungbean. Some mungbean genotypes also showed superior heat stress response, which were further validated using key candidate genes involved in the physiological processes. Results obtained from this study are expected to be useful in breeding strategies aimed at developing heat-tolerant mungbean varieties, thereby contributing to sustainable agricultural practices and food security in regions prone to heat stress.

## 2 Materials and methods

### 2.1 Plant material and field trial

Plant materials comprised of 87 mungbean genotypes, which were grown during the summer season in the first week of April 2022, when day/night temperatures at the reproductive stage were >40/28 C (sown in such a way that the reproductive stage coincided with heat stress periods). The experiment was conducted at the National Bureau of Plant Genetic Resources, New Delhi, India (28° 34′47″N; 76° 50′52″E) India. The basic passport information on tested mungbean genotypes is presented in Table 1. The mungbean genotypes used in this study were obtained from ICAR-NBPGR Gene Bank in New Delhi, India. Genotypes were selected with similar maturity duration (70–75 days in this experiment), so exposure of heat stress is synchronized at the reproductive stage of each genotype. The field experiments were done in a randomized complete block design (RCBD) in two replications in each season. Four check mungbean varieties *viz.*, SML668, IPM-99-125, Pusa Vishal and Virat were used. These varieties were selected as checks in this experiment as these are known for their heat stress tolerance nature and are recommended for summer cultivation in different parts of India (Tiwari and Shivhare, 2019). Each plot consisted of double rows of 3 m in length. Rows were spaced at 30 cm and interplant distance was 10 cm. Two trials were conducted during the “summer season (SS)” and “rainy season (RS).” The heat tolerance of mungbean germplasm was determined by measuring leaf area, chlorophyll content, MSI, pollen viability, number of pods per cluster, 100 seed weight, number of pods/plant, number of seeds/pod and grain yield/plant.

**TABLE 1:** List of accessions used in this study with biological status, source, and collection date.

Sr No.	Accession no.	Collector no.	Other identity	Biological status	Source	Collection date
1	IC616107	PI 376998	VI000578AG	Local germplasm	Bihar	26-01-1973
2	IC121301	PLM-962		Local germplasm	Unknown	
3	IC252010			Local germplasm	Unknown	16-08-1999
4	IC8917		Maru-15	Local germplasm	Rajasthan	19-12-1961
5	IC623923	PI363504	MING 6	Breeding Line	Kerala	18-05-1970
6	IC616240	PI 305413	VI003907AG	Local germplasm	Unknown	01-01-1976
7	IC11365			Local germplasm	Gujarat	25-11-1964
8	IC623822		LGG 460	Released Variety	Andhra Pradesh	18-08-2017
9	IC394728	SMBR-611	Sona Mung	Landrace	Asam	22-03-2003
10	IC616201	PLM-576	VI003455AG	Local germplasm	Punjab	01-02-1976
11	EC862623	PI 425842	VI002487AG	Local germplasm	Pakistan	01-12-1975
12	IC623939	PLM 1041	PI364187	Breeding Line	Unknown	18-05-1970
13	IC9137-1		Maru-4	Local germplasm	Rajasthan	20-12-1961
14	IC616225	PLM-949	VI003720BG	Local germplasm	Unknown	
15	RMG-344			Released Variety	Rajasthan	
16	IC488823	PLM-350		Local germplasm	Unknown	
17	IC103821	DCB-1287		Local germplasm	Gujrat	14-10-1989
18	IC488554	PLM-578	PI 363802	Local germplasm	Punjab	02-12-1965
19	IC623945	II-277-97	PI377014	Breeding Line	Bihar	12-07-1971
20	IC118998		COGB-2	Local germplasm	Punjab	
21	IC616101	M-560	VI000532BG	Local germplasm	Bihar	26-01-1973
22	IC76585	M-942		Local germplasm	Delhi	31-12-1967
23	IC39293		47/1	Local germplasm	Rajasthan	12-08-1980
24	IC489101	PLM502	PI 363742	Local germplasm	Punjab	02-12-1965
25	EC398937		VC 6173B-14	Local germplasm	Thailand	31-09-1996
26	IC488879	PLM-324		Local germplasm	Bihar	
27	IC76471	M-653		Local germplasm	Delhi	31-12-1967
28	IC9125	PI363223		Local germplasm	Rajasthan	20-12-1961
29	IC553601		UPM 02-17	Released Variety	Uttarakhand	30-07-2007
30	IC507459	PLM-645		Local germplasm	Gujrat	
31	IC314609	PLM-666		Local germplasm	Gujarat	01-03-2000
32	IC488778	PLM-89		Local germplasm	Unknown	
33	IC488573	PLM-66	PI 363375	Local germplasm	Unknown	02-12-1965
34	IC73532	ML-267		Local germplasm	Punjab	01-03-2000
35	IC251553	V - 2808		Local germplasm	Taiwan	15-06-1988
36	IC488904	PLM-634		Local germplasm	Punjab	
37	IC489114	PLM-424		Local germplasm	Haryana	
38	EC396116			Local germplasm	Unknown	
39	IC313547	NDS-47	Pesalu	Local germplasm	Bihar	10-02-2001
40	IC507299-2	PLM-126	PI 363431	Local germplasm	Bihar	02-12-1965
41	IC314666	PLM-0743		Local germplasm	Gujrat	01-03-2000
42	IC507517	PLM-849	PI 364028	Local germplasm	Rajasthan	01-03-2000
43	IC548275	WGG 37	ULG-37	Local germplasm	Unknown	
44	IC148531	SV-2438		Local germplasm	Maharashtra	
45	IC507342	PLM-234		Local germplasm	Bihar	
46	IC548274	WGG 2	ULG-2	Local germplasm	Telangana	01-01-2006
47	IC76475	M-658		Local germplasm	Delhi	31-12-1967
48	IC325788	KCM/BR-72		Local germplasm	Rajasthan	12-10-2001
49	IC488775	PLM-169	PI 363470	Local germplasm	Unknown	02-12-1965
50	IC148530	N-1535		Local germplasm	Maharashtra	
51	IC76422	M-553		Local germplasm	Unknown	
52	IC489062	PLM-775	PI 363972	Local germplasm	Rajasthan	01-03-2000
53	IC76460	M-633		Local germplasm	Delhi	31-12-1967
54	IC507415	PLM-483	PI 363725	Local germplasm	Karnataka	
55	IC314272	PLM-0012		Local germplasm	Uttar Pradesh	01-03-2000
56	IC548266		LGG 450	Released Variety	Andhra Pradesh	01-01-1995
57	IC121190	PLM-180		Local germplasm	Unknown	
58	IC12120	PLM-252		Local germplasm	Uttar Pradesh	02-12-1965
59	IC488532	PLM-514	PI 363752	Local germplasm	Punjab	02-12-1965
60	EC251786		VC - 3004 A	Local germplasm	Taiwan	16-06-1988
61	IC309457	ML-1	V-2772	Local germplasm	Taiwan	07-09-1990
62	IC623914	MARU 10	PI363230	Breeding Line	Rajasthan	
63	IC507310	PLM-161		Local germplasm	Bihar	
64	IC616215	PLM 721	VI003563A-BR	Local germplasm	Gujrat	18-05-1970
65	IC616138	PI 425324	VI001562AG	Local germplasm	Unknown	09-04-1973
66	IC52078	TR 276/5		Local germplasm	Haryana	08-03-1982
67	IC119106		Lihulst-P1	Local germplasm	Maharashtra	
68	IC76338	M-401	Mung bean	Local germplasm	Unknown	
69	IC398984	SK-37	Pesaralu	Landrace	Andhra Pradesh	19-02-2003
70	IC76389	M-487	Mung bean	Local germplasm	Unknown	
71	IC507463	PLM-653	PI 363859	Local germplasm	Gujrat	02-12-1965
72	IC418452		VC 6153B-20P	Local germplasm	Andaman and Nicobar Island	21-11-2003
73	EC398882			Local germplasm	Thailand	31-09-1996
74	IC507293	PLM-118		Local germplasm	Bihar	
75	EC2851-3			Local germplasm	Uttarakhand	21-07-1969
76	EC251557		V - 6,017	Local germplasm	Thailand	15-06-1988
77	IC507321	PLM-188	PI 363487	Local germplasm	Uttar Pradesh	02-12-1965
78	IC76479	M-665		Local germplasm	Delhi	31-12-1967
79	IC121299	PLM-959		Local germplasm	Unknown	
80	IC398901		VC 6153B-19	Local germplasm	Thailand	31-09-1996
81	IC76583	M-940		Local germplasm	Delhi	31-12-1967
82	IC305249			Local germplasm	Unknown	
83	Chait mung			Landrace	West Bengal	30-03-2019
84	IC623821		SML-668	Released Variety	Unknown	18-08-2017
85	IC623703		IPM 99-125	Released Variety	Uttar Pradesh	04-08-2017
86	IC589309	IPM 205-7	VIRAT	Released Variety	Uttar Pradesh	18-08-2011
87	IC623705		Pusa Vishal	Released Variety	Delhi	04-08-2017

### 2.2 Meteorological data

**FIGURE 1:**
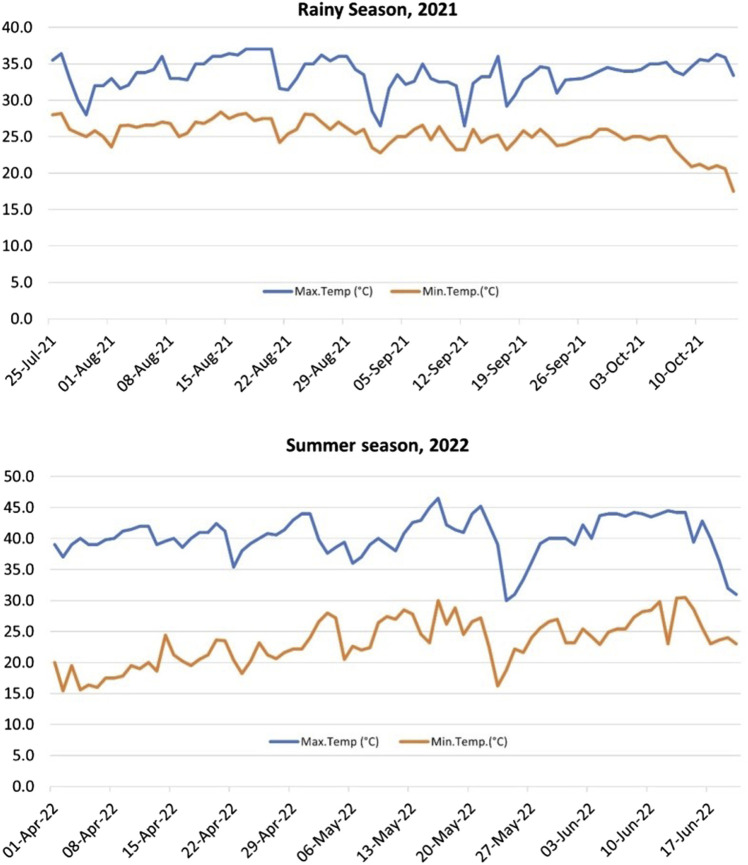
Atmospheric temperature profile for the rainy season (RS), 2021 and summer season (SS), 2022 of the experiments. The average maximum day temperature for SS was significantly higher (40.3°C) than the RS (33.6°C).

Weather data for the New Delhi Area location for both the seasons *viz.* RS and SS was recorded throughout the growing period by the meteorological observatory available at the location (Supplementary Material; Figure 1).

### 2.3 Physio-morphological traits

The morphological and physiological parameters were taken at the appropriate plant growth stage, between flowering initiation to maturity of genotypes. To measure leaf parameters such as leaf area, chlorophyll content, membrane stability index (MSI) and stomatal conductance, young and fully developed leaves were marked in each genotype. Similarly, for pollen viability assessment fully developed flower buds about to open were selected. To measure the number of pods per cluster the central branch of three plants from each genotype were selected. Three random plants were used to count number of pods/plant and number of seed/pod. 100-seed weight and grain yield/plant were other morphological parameters estimated from each replication. The SPAD chlorophyll meter reading (SCMR) was used as an indicator of leaf chlorophyll content. The SPAD value of a marked leaf in each line was recorded between 10.00 and 11.00 a.m., using an Apogee-SPAD meter. The stomatal conductance was measured using SC-1 Leaf Porometer (METER Group, Inc.). The membrane stability index (MSI) was calculated using the method given by Lutts et al. (1996). In this process, three young leaves were collected from all genotypes is thoroughly washed with distilled water. The leaf tissues are then placed in 10 mL of distilled water at 40°C for 30 min and electrical conductivity readings are recorded (C1) using a conductivity meter. The same samples are subsequently subjec€ted to boiling water at 100°C for 10 min and readings are recorded again (C2). The percentage of membrane stability index (MSI %) is calculated using the formula $MSI\%=\left[1-\left(\frac{C1}{C2}\right)\right]\times 100$ with the units expressed as a percentage. For the examination of pollen viability, the percentage of viable pollen grains is determined using either a 0.5% acetocarmine solution or the Alexander triple stain (ATS) solution described by Alexander (1969). Heat susceptibility index (HSI) was estimated for seed yield/plant using the formula $HSI=1-\left[\frac{Ysi}{Ypi}\right]/1-\left[\frac{Ys}{Yp}\right]$ as suggested by Fischer and Maurer (1978). Where, Ysi and Ypi are yield/plant under heat stress and control conditions respectively, while Ys and Yp are the mean yield of all genotypes under heat stress and control conditions.

### 2.4 Validation of promising heat stress tolerant mungbean genotypes under an artificially controlled environment using qRT-PCR

Seven mungbean genotypes were selected based on the field evaluation data on morphological and physiological performance under heat stress (SS) and normal control (RS) environments. Seven mungbean genotypes used for key candidate gene expression analysis using qRT-PCR analysis included three heat tolerant (IC76475, IC418452, IC489062), two heat-sensitive genotypes (IC616138, IC548275) and two control check cultivars (SML668, IPM99-125). The check varieties SML-668 and IPM 99-125 were selected as they were found superior among all the four check varieties used in this study. These genotypes were grown in plastic pots (8 inch radius) containing soil and fertilizer mix. Randomized complete block design was used for both heat-treated and control plants. The experiment was conducted in the control environment chambers of the National Phytotron Facility of ICAR-IARI, New Delhi. The initial growth conditions were set as 28°C temperature and 16-h light/8-h dark photoperiod conditions. After 3 weeks of the growth period, heat stress treatment of 40°C was given. The day temperature was 40°C and the night temperature was 28°C. The leaf samples from both treatments were collected in liquid nitrogen for the candidate gene expression analysis.

### 2.5 RNA isolation, cDNA synthesis and qRT-PCR analysis

Total RNA was isolated from treated and untreated mungbean seedling samples using the TRIAZOL method. The first strand synthesis was carried out from control and stressed plant samples using a Thermoscientific RevertAid first-strand cDNA synthesis kit. The samples were mixed through pipetting and centrifuged briefly in a micro-centrifuge, then tubes were incubated in a thermal cycler for 1 h at 42°C. After finishing the reaction time, tubes were immediately kept on ice for termination of the reaction. Later, 30 µL RNase free water was added to dilute the cDNA and stored at −20°C or instantly started the second-strand cDNA synthesis. The primers for key candidate genes known to play a significant role in heat stress tolerance were taken from the published research works (Table 2).

**TABLE 2:** List of primers of key candidate genes used for the analysis of their expression using qRT-PCR.

Gene name	Primers		Function	References
CP47	F	TGG​GTG​TCT​GAT​CCT​TAT​GGA​C	Photosystem II CP47 reaction centre protein, Chlorophyll biosynthesis	Paul et al. (2018)
	R	CCT​GCC​GCA​ATA​TGA​TGA​GAA​G		
SUT	F	TGT​CTC​CGT​CAC​AGC​ACC​AC	Sugar efflux transporter, Pollen development	Paul et al. (2018)
	R	GGA​GGA​GCC​AAC​CAA​GTT​ACA​G		
MTR_1g062190	F	ACC​ACT​GGT​TCG​GTG​CTT​AC	Cytochrome P450 monooxygenase, cellular metabolism and homeostasis	Singh et al. (2019)
	R	AGC​TCA​TCG​GGA​ACT​TGA​GA		
MTR_7g092380	F	TCG​CTT​TGA​TTG​CTT​TGA​TG	DNAJ chaperone, protein homeostasis	Singh et al. (2019)
	R	CAT​ATC​ACA​ACG​CCG​AAA​T G		
LOC101499292	F	TTC​TCT​CCA​ACA​CGG​AGC​TT	HSP 22 KDa-Mitochondria based	Singh et al. (2019)
	R	AGT​CCA​GGC​ATA​TCC​AAA​CG		
VrLEA-1	F	CCT​CGG​ACA​GAA​ACA​ACC​GA	*Vigna glabrascens* ATP synthase beta subunit (ATPβ) gene	Singh et al. (2022)
	R	GAC​CCG​GTA​CTG​AGG​GGT​AT		
Actin	F	ATG​GTG​GGT​ATG​GGT​CAA​AA	References gene	Zhang et al. (2020)
	R	GAG​GAC​AGG​ATG​CTC​CTC​AG		

The key candidate genes involved in heat stress defense mechanisms were identified through the literature search based on the criteria of their potential roles in regulating physiological and phenotypic traits in *Vigna* or any other legume crops. From this, six key candidate genes were selected for qRT-PCR analysis, meeting the criteria of relevance and successful PCR amplification (Table 2). The gene expression quantification was done using SYBR^®^ Premix kit in Light Cycler 480^®^ Real-Time PCR System (CFX96 Real-time PCR system). For each sample, three biological replicates were used and three technical replicates were kept for each biological replicate. Actin was used as a housekeeping gene for the RT-PCR. The thermal cycler conditions included initial denaturation at 95°C for 2 min and 40 amplification cycles. Each amplification cycle included denaturation at 95°C for 20 s, primer annealing at 60°C for 35 s, and extension at 72°C for 30 s. To identify non-specific amplification the melt curve analysis was done with a gradual temperature increase of 1°C/s from 60°C to 95°C. Analysis of qRT-PCR data was carried out based on the quantification of cycle (Cq) values from each well. The resulting data was calculated by the 2^-(ΔΔCT)^ method (Schmittgen and Livak, 2008). The log2-fold change of 2^-(ΔΔCT)^ values used to quantify the gene expression in response to heat stress treatments for each candidate gene. A t-test was used to calculate statistical significance using SPSS software (Park, 2009). The qRT-PCR results were used to compare the expression level of six key candidate genes (listed in Table 2). The enhanced expression of these genes helps plants to combat heat stress. Therefore, these genes were used to validate their role in the selected mungbean genotypes including three heat tolerant, two susceptible, and two check varieties of mungbean. The qRT-PCR results helped in the validation of mungbean genotypes as heat stress tolerant.

### 2.6 Statistical analysis

Microsoft Excel was used for arranging the raw data and conducting calculations for the mean and standard deviation of both the control group and each treatment trait. The statistical analysis in this research was performed using R version 4.0.4 and SAS 9.4 software. For cluster analysis package “cluster” used which facilitates in various cluster analysis, including hierarchical clustering. The “gplots” R package enhances the graphical representation of data, offering functions for constructing dendrograms. Other packages include “corrplot” and “Hmisc” for correlation studies. The “FactoMineR” and “factoextra” packages are instrumental in creating biplots, particularly in principal component analysis (PCA). In SAS, the “proc glm” procedure used for analysis of variance (ANOVA), providing an ANOVA table to assess group mean differences. The “means” statement with the “tukey” option in “proc glm” used for Tukey *post hoc* test, generating *p*-values for pairwise comparisons, essential for identifying significant differences among treatment groups.

## 3 Results

### 3.1 Physiological changes of mungbean genotypes under heat stress

**FIGURE 2:**
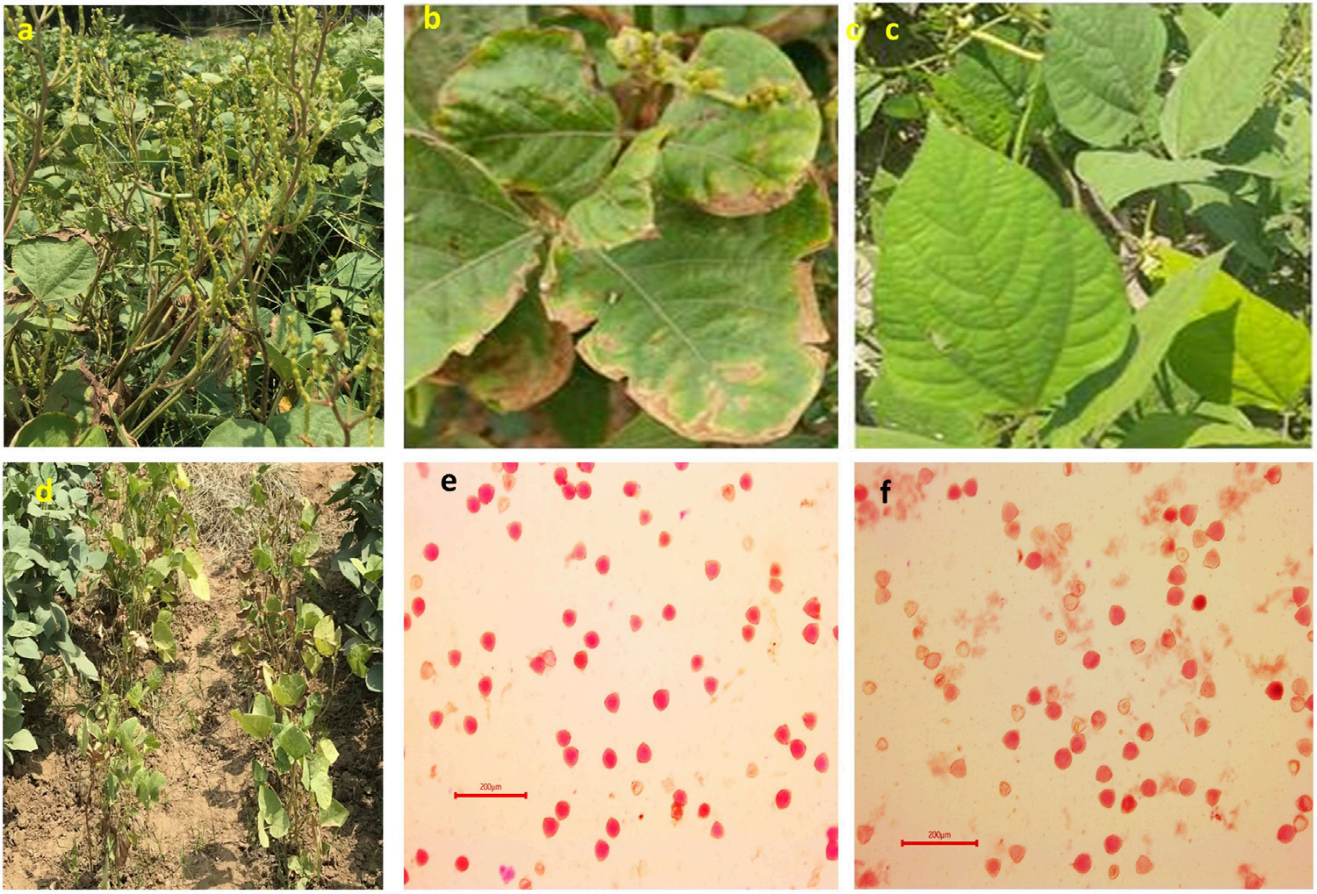
Typical morphological symptoms in mungbean plants in response to heat stress for various mungbean plant parts such as severe flower drop in susceptible mungbean genotype **(A)**; SML668, a check variety used in this experiment showed leaf margin burning **(B)** under SS condition, and no symptom of leaf burning **(C)** under RS conditions; effect of heat stress on heat-sensitive genotypes showing poor plant growth and leaf burning **(D)** under SS conditions. Photographs showing comparative higher pollen viability in IC76475, a tolerant mungbean genotype **(E)**, and low pollen viability in IC616138, a susceptible genotype **(F)** under SS conditions.

A significant amount of variation in response to heat stress was observed for morphological and physiological characteristics such as number of pods/plant, number of pods/cluster, number of seed/pod, MSI, chlorophyll content, pollen viability %, 100 seed weight (Table 3; Figure 2). The promising genotypes under RS and SS conditions superior to the check variety are shown in Table 3).

**TABLE 3:** Summary statistics of morpho-physiological parameters and promising mungbean genotypes under RS and SS conditions.

Parameters	Condition	Min	Max	Mean	Std. Dev	CV % (P)	Promising genotypes	Best check
Leaf Area (cm^2^)	RS	26.65	83.91	47.89	12.28	25.65	—	
	SS	17.88	65.96	37.79	8.11	21.46	—	
No. of pods/plant	RS	21.00	91.50	49.95	17.05	34.14	IC39293 (91.5), IC616215 (91.5), IC488823 (84.5), IC616138 (84.0), IC398984 (84.0)	Pusa Vishal (73.0)
	SS	15.50	59.00	33.03	9.40	28.44	IC489062 (59.0), IC252010 (55.5), IC488778 (47.50), IC418452 (47.50)	IPM-99-125 (47.5)
No. of pods/cluster	RS	1.83	12.58	5.89	2.28	38.64	IC252010 (12.58), IC398984 (11.25), EC862623 (10.67), IC616201 (10.50), IC76475 (10.33)	SML-668 (9.67)
	SS	2.50	8.67	4.88	1.34	27.51	IC76475 (8.67), IC52078 (8.00), IC418452 (7.92), IC507321 (7.83), IC507310 (7.25), EC251786 (7.17), IC488532 (7.17), IC76389 (7.00), IC507463 (6.83), IC489062 (6.67)	IPM-99-125 (4.5)
No. of Seeds/pod	RS	7.00	14.00	10.62	1.46	13.78	IC398901 (14.00), IC76422 (13.33), IC623822 (13.33), EC398882 (13.00), IC507342 (12.67)	Pusa Vishal (12.0)
	SS	4.83	11.67	7.11	1.43	20.05	IC76475 (11.67), IC418452 (10.17), IC507517 (9.83), IC76585 (9.50), IC488879 (9.33), IC507299-2 (9.33), IC488554 (9.33), IC76471 (9.17)	Pusa Vishal (8.0)
MSI	RS	26.48	81.25	58.22	11.87	20.38	IC616215 (81.25), IC76389 (80.12), IC52078 (76.96), IC488573 (75.92), IC76585 (75.72)	Pusa Vishal (69.3)
	SS	65.75	90.19	83.02	5.19	6.25	IC148530 (90.19), IC418452 (89.17), IC76475 (88.92), EC251557 (88.59), IC489062 (88.53)	IPM-99-125 (89.23)
CC (micromole/m^2^)	RS	15.45	45.70	32.24	6.06	18.78	IC313547 (45.70), IC616138 (44.10), IC488879 (43.35), IC148530 (41.35), IC309457 (41.00)	Pusa Vishal (31.95)
	SS	13.45	35.55	22.75	5.08	22.35	IC489062 (35.55), IC418452 (34.40), IC121299 (33.25), IC507293 (31.25), IC73532 (31.05), IC616225 (30.55), IC553601 (30.00), IC314272 (29.90), IC623923 (29.80), IC76475 (29.75)	IPM-99-125 (23.65)
CT (°C)	RS	24.25	32.69	29.26	1.61	5.50	IC616107 (24.25), IC121301 (24.78), IC616240 (25.86), IC488879 (25.89), IC76471 (25.94)	SML-668 (27.28)
	SS	27.95	34.65	31.21	2.29	7.35	EC862623 (27.95), IC121301 (28.20)	SML-668 (28.3)
CTD (°C)	RS	3.81	11.89	6.72	1.73	25.79	IC616107 (11.89), IC121301 (11.36), IC488879 (11.06), IC76471 (11.00), IC616240 (10.28)	SML-668 (8.69)
	SS	7.62	27.87	11.47	2.49	21.68	IC309457 (14.62), IC314272 (14.37), IC103821 (14.20), IC121301 (14.18), EC862623 (14.10)	SML-668 (13.75)
Pollen viability (%)	RS	42.27	93.27	79.60	9.61	12.07	IC548274 (93.27), IC39293 (92.12), IC623822 (91.97), IC489062 (91.61), IC507459 (91.04)	Pusa Vishal (87.8)
	SS	7.78	100.00	62.16	20.29	32.64	IC418452 (100.0), IC314272 (98.15), IC314609 (95.45), IC313547 (92.56), IC507299-2 (90.28)	Pusa Vishal (86.96)
100 Seed weight (g)	RS	1.50	6.36	3.73	0.72	19.39	EC251557 (6.36), IC12120 (5.25), IC507517 (5.01), IC548266 (4.94)	SML-668 (4.93)
	SS	1.80	6.78	3.06	0.72	23.47	IC418452 (6.78), IC507321 (5.06), IC489062 (4.19)	SML-668 (5.31)

Abbreviations: CT, canopy temperature; CTD, canopy temperature depressions; CC, chlorophyll content; MSI, membrane stability index.

**FIGURE 3:**
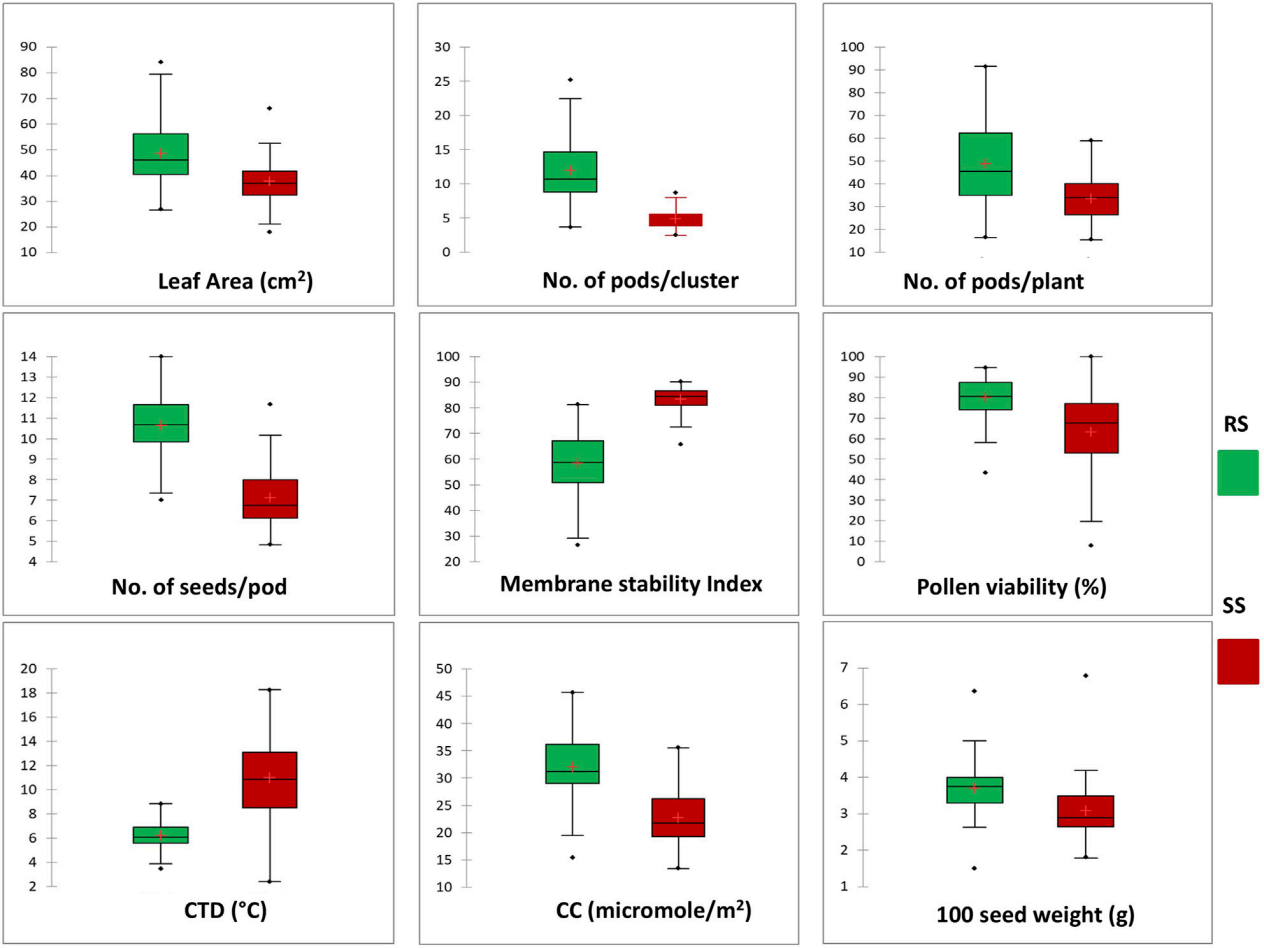
Box plot chart distribution of morphological and physiological traits under RS and SS conditions. The distribution for each trait shows a significant change in the phenotypic expression of mungbean genotypes in response to heat stress.

Significant deviations in morphological characteristics are observed across the mungbean genotypes under heat stress (SS) in comparison to normal sown (RS) environment conditions (Figure 3). Although a significant amount of variation was observed for the traits studied in both the environments, i.e., RS and SS, a reduction in the amount of variation is observed in the SS environment for all the traits, except for the number of seeds/pod and pollen viability % (Table 3). It is also observed that each genotype follows its own distinct mechanism of heat stress tolerance, as observations on multiple parameters revealed that no individual genotype is superior for all the parameters (Table 3). However, superior genotypes are observed for most of the traits. A notable reduction in leaf area, along with the appearance of associated morphological symptoms such as wilting and scorching was observed in the SS environment. Under RS condition the higher leaf areas were recorded in mungbean genotypes *viz.*, IC103821 (83.90 cm^2^), IC623914 (79.54 cm^2^) and IC9125 (78.31 cm^2^). While, in SS condition the maximum leaf areas were observed in mungbean germplasm lines *viz.*, IC623914 (65.95 cm^2^), IC507415 (57.78 cm^2^) and IC418542 (52.68 cm^2^). Among check varieties, SML-668 exhibited superior performance in both seasons for the leaf area. Similarly, superior mungbean germplasm lines are identified for other traits also as mentioned in table 3.

The genotype IC418452 excelled among all the genotypes under the heat stress (SS) environment (Table 3), although its performance under normal conditions (RS) was not significant. The genotype has shown excellent heat stress adaptability for traits *viz.*, number of pods/plant, number of pods per cluster, number of seeds/pod, MSI (%), chlorophyll content, CT, CTD, and pollen viability and also retained grain weight. Another genotype IC489062 has also shown good adaptability for heat stress tolerance as revealed by its superior performance for traits *viz.*, number of pods/plant, number of pods/cluster, MSI, chlorophyll content and seed weight. The passport information indicates that the genotype IC418452 originated from the Andaman and Nicobar Islands, while the genotype IC489062 belonged to Rajasthan (Table 1). The genotype IC76475 was found superior for multiple traits *viz.*, number of pods/plant, number of pods/cluster, number of seeds/pod and MSI (%) under SS conditions. The genotype IC314272 is found superior for canopy temperature and canopy temperature depression. IC314272 showed heat stress adaptability through better CTD, higher pollen viability and better chlorophyll content. The genotype EC862623 was found superior for traits *viz.*, number of pods/cluster, CT and CTD. There is another group of genotypes that have excellently performed in terms of their adaptability for a single or two traits (Table 3). Some genotypes have performed relatively better under the control environment (RS), which can find their direct use in mungbean trait improvement programs other than the heat stress (Table 3). The stomatal conductance (mol m⁻^2^ s⁻^1^) showed variation ranging from 2.87 to 10.93 mol m⁻^2^ s⁻^1^ under SS conditions. The check varieties *viz.*, SML-668 (4.10), IPM 99-125 (4.83), Virat (4.680) and Pusa Vishal (4.00) showed moderate levels of stomatal conductance. The promising mungbean genotypes *viz.*, IC489062 (5.30), IC76475 (4.33) and IC418452 (4.07) also showed moderate levels of stomatal conductance. This indicates that a plant needs to adapt to heat stress conditions by evaporation to cool down its canopy, while it also must maintain desired leaf water potential to maintain turgor pressure, gaseous exchange, photosynthesis and other physiological activities.

Ranking of 87 mungbean genotypes based on their heat susceptibility index (HSI) indicated a good range of variability for heat stress tolerance in terms of their yield performance under a heat stress environment. The genotypes *viz.*, IC623822, IC616201 and IC489062 were found superior to the best mungbean check variety, i.e., SML-668 (Table 4).

**TABLE 4:** Categorization and ranking of mungbean genotypes’ relative susceptibility to heat stress.

Sr No.	Genotypes	HSI^a^	Sr No.	Genotypes	HSI
1	IC616138	1.23	45	IC394728	1.04
2	IC488532	1.23	46	IC76422	1.04
3	IC507463	1.23	47	IC488554	1.04
4	IC52078	1.22	48	IC76479	1.01
5	IC309457	1.22	49	IC507310	1.01
6	IC148530	1.21	50	IC488573	1.01
7	IC398984	1.21	51	IC623914	1.00
8	IC76389	1.21	52	IC11365	0.99
9	IC76460	1.20	53	IC488778	0.99
10	IC507415	1.20	54	IC103821	0.98
11	IC148531	1.19	55	IC616225	0.96
12	IC12120	1.19	56	IC507342	0.96
13	EC398882	1.19	57	IC616240	0.96
14	IC548266	1.18	58	IC314666	0.95
15	IC313547	1.17	59	IC489114	0.95
16	IC76585	1.17	60	IC507321	0.94
17	IC73532	1.16	61	IC418452	0.92
18	RMG-344	1.16	62	IC623945	0.91
19	IC9137-1	1.16	63	Pusa Vishal	0.90
20	EC396116	1.16	64	IC553601	0.89
21	IC305249	1.15	65	IC488879	0.88
22	IC121299	1.15	66	Chait mung	0.86
23	IC507459	1.15	67	IC616101	0.86
24	EC251557	1.15	68	IC39293	0.85
25	IC314272	1.14	69	IC507517	0.80
26	IC623939	1.14	70	IC325788	0.78
27	EC2851-3	1.14	71	IC623923	0.74
28	IC507293	1.14	72	IC548274	0.73
29	IC314609	1.13	73	IC488823	0.69
30	IC489101	1.12	74	IC488904	0.68
31	IC488775	1.11	75	IC76475	0.67
32	EC251786	1.11	76	IC252010	0.67
33	EC398937	1.10	77	IC8917	0.64
34	IC76471	1.10	78	Virat	0.63
35	IC548275	1.10	79	IC616107	0.60
36	IC76338	1.09	80	IC121301	0.48
37	IC119106	1.09	81	IC118998	0.40
38	IC616215	1.08	82	IC507299-2	0.37
39	IC76583	1.07	83	IC251553	0.35
40	IPM 99-125	1.07	84	SML-668	0.32
41	IC121190	1.07	85	IC623822	0.20
42	IC9125	1.06	86	IC616201	0.16
43	IC398901	1.06	87	IC489062	−0.10
44	EC862623	1.05			

^a^ HSI, Heat Susceptibility Index. Note: A higher index value indicates a higher susceptibility of genotype to heat stress.

### 3.2 Correlation analysis and ANOVA

**FIGURE 4:**
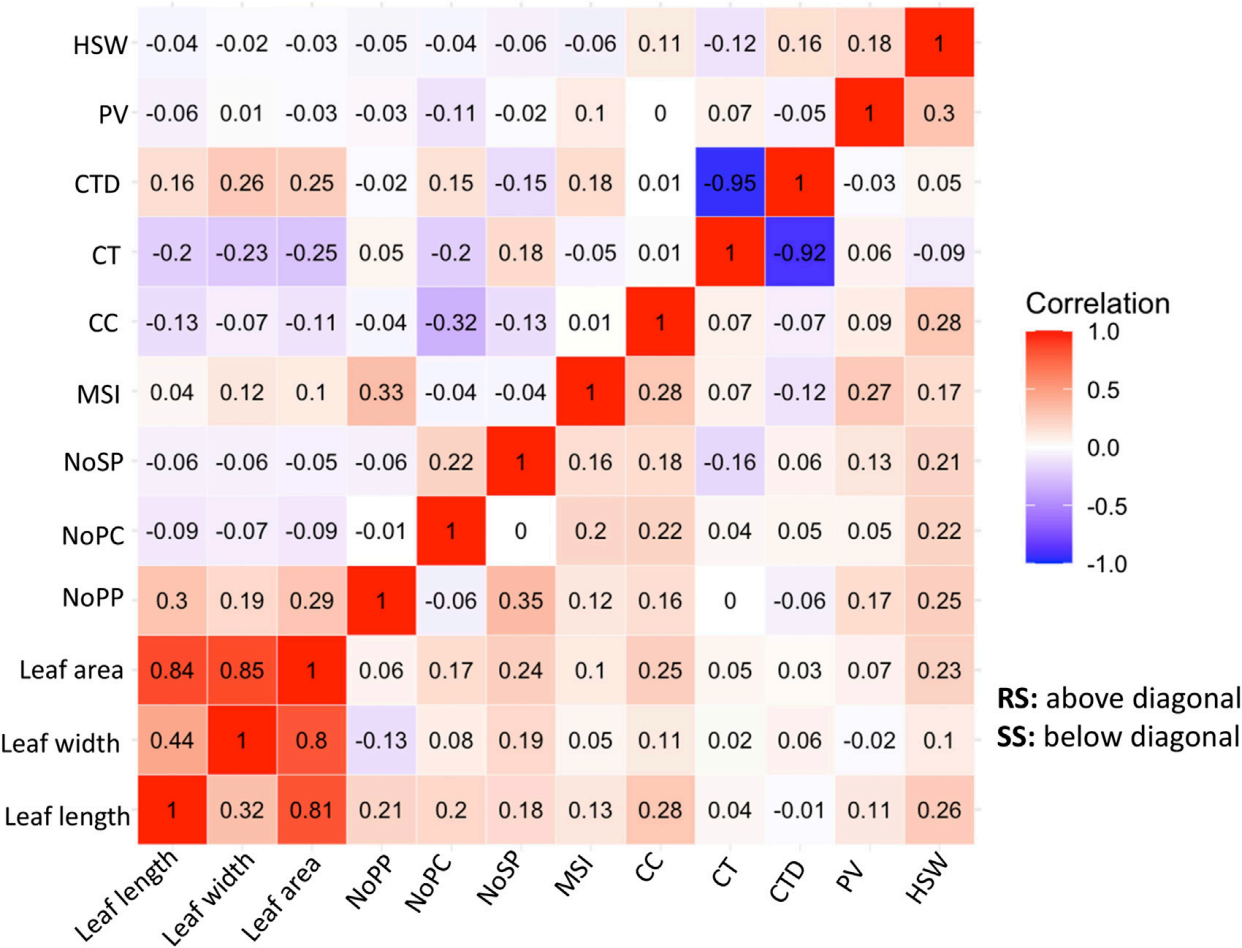
Correlation Matrix of morpho-physiological traits of 87 mungbean genotypes under normal sown conditions (RS) and heat stress (SS) environments showing character correlation with each other [A positive correlations are shown in red and negative correlations are shown in blue color. The color intensity indicates the magnitude of correlation in either direction].

Correlation analysis is a statistical method used to evaluate the relationship between two variables. In the context of plant physiology and morphology, correlation analysis can help uncover their relationships with the adaptability of the genotypes. In this study the correlations among the physiological and morphological parameters under SS and RS conditions were examined (Figure 4). The correlation analysis showed the important relationships between the morphological and physiological parameters studied under RS and SS conditions. A strong and negative correlation is observed between canopy temperature (CT) and canopy temperature depression (CTD), which indicates the efficiency of mungbean genotypes to cool down the canopy temperature through transpiration (Figure 4). Positive correlations were observed among leaf area, leaf length and leaf width. The leaf dimension traits have shown moderately negative correlations with CT under RS conditions, while the correlations are negligible under SS conditions (Figure 4).

ANOVA was done to understand the differences among the genotypes under the two distinct growing conditions, i.e., SS and RS with respect to morpho-physiological parameters. The ANOVA indicated that the expressions of traits *viz.*, leaf area, number of pods/cluster, number of pods per plant, seed per pod, pollen viability, canopy temperature, chlorophyll content, 100 seed weight and membrane stability index under rainy season (RS) and summer season (SS) environments are significantly different at the given significance level (α) of 0.0001 (Table 5). Overall, ANOVA proved to be a valuable statistical tool for detecting the impact of treatment on morpho-physiological traits in plants.

**TABLE 5:** ANOVA for morpho-physiological parameters under SS conditions (heat stress) than the RS conditions (normal) of 87 mungbean genotypes.

Parameters	Leaf area	Pods/bunch	Pod/Plant	Seed/Pod	100 SW	MSI	PV	CT	CTD	CC
Genotype mean square	259.8	7.6	574.5	4.2	19.1	165.5	629.6	11.4	10.6	61.6
Condition mean square	10,400.4	103.2	25,432.8	1073.9	103.2	53,485.7	23,637.4	364.5	1896.3	7538.6
Error mean square	84.8	4.5	78.2	2.7	18.2	60.7	200.6	7.7	7.5	23.2
Pr > F (Condition)	***	***	***	***	***	***	***	***	***	***
Pr > F (Genotype)	***	***	***	***	***	***	***	***	***	***
TSRT-MSD (Genotypes)	2.93	6.45	26.91	4.97	12.97	23.71	43.08	8.46	8.32	14.64
TSRT-MSD (Condition)	1.94	0.45	1.87	0.34	0.90	1.65	2.99	0.59	0.58	1.02

Abbreviation: ***significance level (α) of 0.0001, 100 SW (100 seed weight), MSI (membrane stability index), PV (pollen viability), CTD (canopy temperature depression), CC (chlorophyll content), TSRT-MSD (Tukey’s Studentized Range Test- Minimum Significant Difference). Conditions are RS, and SS.

### 3.3 Principal component analysis

The Principal Component Analysis (PCA) was employed in this study to reduce the dimensionality of the multivariate dataset and identify underlying patterns in the data. By transforming the original variables into a set of orthogonal principal components, PCA allowed us to capture the most significant sources of variation and extract meaningful information from the complex dataset. In this study, PCA was employed to analyze a dataset of morpho-physiological traits in plants, e.g., leaf area, number of pods/cluster, number of pods/plant, seeds/pod, pollen viability%, chlorophyll content, 100 seed weight and MSI.

**FIGURE 5:**
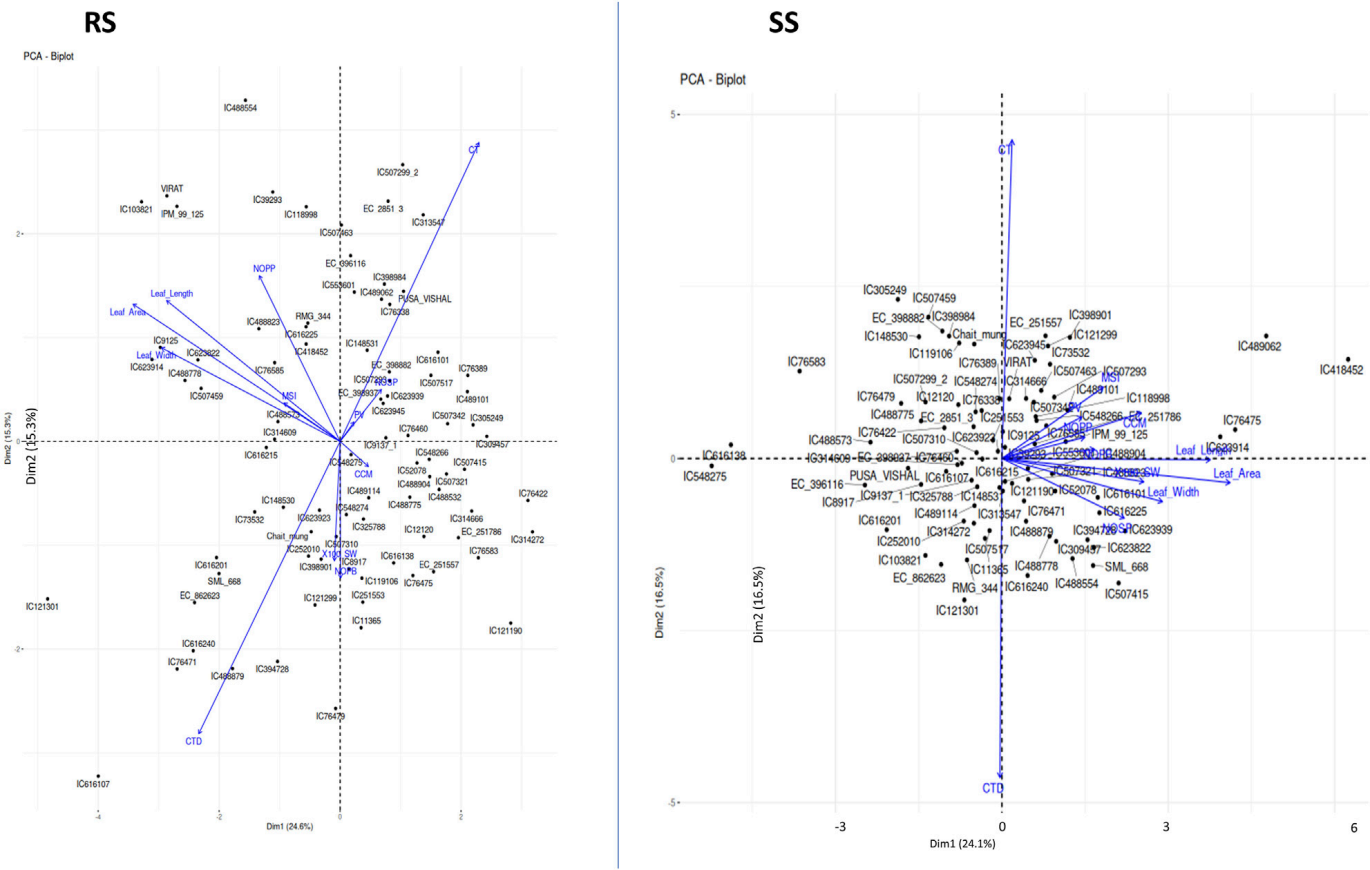
Principal Component Analysis (PCA) plots showing the multivariate variation among 87 mungbean genotypes in terms of environmental variables. The first two PCs explained 39.91% and 40.65% of the total variance under RS conditions and SS conditions. The variables with closer angles are more positively correlated, while variables in opposite directions are negatively correlated. A similar pattern is observed in both environments except for a few variables, which may be due to the partitioning of the variability in other PCs.

The first five PCs could only explain 72% of the entire variability under both environments, i.e., RS and SS (Table 6). Loading values in each PC indicate the major contributing parameter for the variation. For instance, traits related to leaf dimension and CTD are the major contributors of PC1. PC2 captures a contrast between CT and CTD, likely indicating a trade-off between growth parameters and canopy temperature. PC3 focuses on reproductive characteristics such as the number of pods and pod volume, as opposed to leaf area and width. PC4 differentiates primary branch counts and reproductive outputs like NOPP and NOSP. PC5 contrasts pod volume with other growth factors, including CC and NOPP. PC1 accounts for the most variance, followed sequentially by PC2, PC3 and so on. Conversely, during the rainy season, the table shows PCA loadings where PC1 contrasts leaf dimensions and canopy temperature against CT, with higher scores indicating reduced leaf size and canopy temperature. PC2 contrasts CT and NOPP with CTD and NOPB, pointing to a balance between reproductive output and temperature resilience. PC3 differentiates reproductive traits, such as the number of branches and pods, from CC, possibly a stress or nutrient content indicator. PC4 contrasts MSI and NOPP against leaf size, while PC5 contrasts pod volume and seed weight with CC, hinting at a relationship between reproductive productivity and stress or nutrient content. These loading patterns indicate that relationships among plant traits shift between the summer and rainy seasons, as a result of their adaptation efficiency. The Principal Component Analysis (PCA) under stress conditions (SS) revealed that the first two PCs, i.e., PC1 and PC2, accounted for 24.1% and 16.5% of the total variation, respectively, summing to a cumulative variance of 40.6% (Figure 5; Table 6). In contrast, under rainy conditions (RS), PC1 and PC2 explained 24.6% and 15.3% of the variance, respectively, resulting in a slightly lower cumulative variance of 39.9% (Figure 5; Table 6).

**TABLE 6:** The principal components and the variance explained by each component under RS condition.

	PC 1		PC 2		PC3		PC4		PC5	
Condition	RS	SS	RS	SS	RS	SS	RS	SS	RS	SS
Eigen Value	2.96	2.89	1.85	1.99	1.54	1.66	1.21	1.21	1.12	0.92
Variance %	24.6	24.1	15.3	16.5	12.8	13.9	10.1	10.1	9.3	7.7
Cumulative Variance %	24.6	24.1	39.9	40.6	52.7	54.6	62.8	64.6	72.1	72.3
Rotation (n × k)										
Leaf Length	−0.44	0.46	0.26	0.0	0.05	−0.17	−0.19	−0.08	−0.01	0.07
Leaf Width	−0.45	0.35	0.17	−0.09	−0.01	−0.46	−0.15	−0.07	−0.11	0.21
Leaf Area	−0.52	0.5	0.26	−0.05	0.02	−0.38	−0.2	−0.1	−0.08	0.09
NOPP	−0.2	0.18	0.31	0.05	0.0	0.43	0.51	−0.47	0.09	−0.26
NOPB	0.0	0.2	−0.26	0.02	0.58	0.03	0.16	0.65	−0.12	−0.22
NOSP	0.1	0.27	0.1	−0.13	0.43	0.24	−0.02	−0.41	−0.29	−0.12
MSI	−0.14	0.22	0.07	0.15	−0.11	0.3	0.75	0.29	0.02	0.15
CC	0.07	0.31	−0.05	0.1	−0.54	0.17	−0.06	0.21	0.27	−0.49
CT	0.35	0.02	0.56	0.68	0.00	−0.14	−0.02	−0.05	−0.08	0.02
CTD	−0.36	0.0	−0.54	−0.68	−0.03	0.06	0.11	0.13	0.05	0.03
PV	0.04	0.18	0.04	0.09	−0.27	0.38	0.16	0.05	−0.71	0.74
100_SW	−0.01	0.31	−0.22	−0.05	−0.3	0.31	−0.08	0.12	−0.53	0.08

Abbreviation: NOPP, number of pods/plant; NOPB, number of pods/bunch; NOSP, number of seed/pod; MSI, membrane stability index; CC, chlorophyll content; CT, canopy temperature; CTD, canopy temperature depression; PV, pollen viability %; 100_SW, 100 seed weight.

These results indicate that the variation explained by the first two PCs under SS conditions is slightly higher compared to RS conditions. This suggests that under stress, the dominant physiological traits that contribute to variance are relatively more pronounced, thereby capturing more of the total variability in fewer components.

### 3.4 Agglomerative hierarchical clustering (AHC)

Cluster analysis was employed to classify mungbean lines based on their morpho-physiological characteristics during both the rainy season (RS) and the summer season (SS). The dendrogram visualizes the hierarchical clustering of a dataset with items color-coded to indicate different clusters.

**FIGURE 6:**
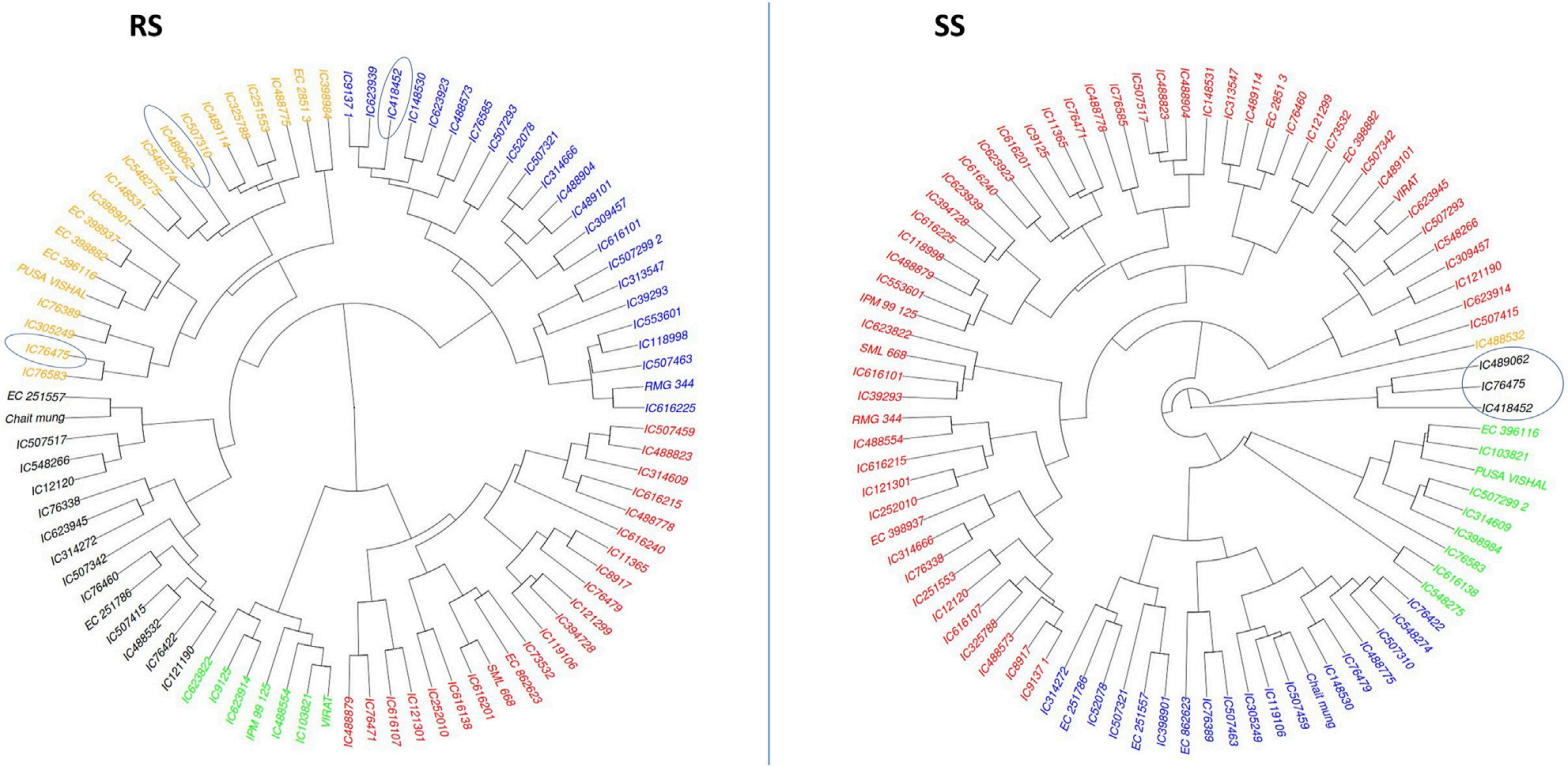
The agglomerative hierarchical clustering of 87 mungbean genotypes under RS and SS conditions. The promising accession identified as promising for heat stress tolerance based on their expression for multiple traits are circled.

In RS, the genotypes are primarily divided into four clusters and a similar clustering pattern is observed in SS. Notably, the differentiation between clusters was more pronounced under SS conditions, indicating greater divergence among genotypes in response to heat stress. Cluster (black colour) under SS conditions, which has three genotypes *viz.*, IC76475, IC489062 and IC418452, are found as heat tolerant based on various morpho-physiological characters. Out of these three genotypes, IC76475 and IC489062 also clustered together in RS conditions, while the genotype IC418452 clustered separately (Figure 6). This indicates that hierarchical clustering is helpful in identification of the similar genotypes for heat stress tolerance, as the tolerant genotypes generally follow similar patterns of trait expression.

### 3.5 Expression analysis of key candidate genes

The expression study of selected key candidate genes was done to validate the results obtained through morpho-physiological work. Here six key candidate genes *viz.*, *CP47, SUT, MTR_1g062190, MTR_7g092380, LOC101499292* and *VrLEA-1* which are known to control important cellular processes as mentioned in table 2 were analyzed for their level of expression under normal temperature and elevated temperature conditions as mentioned in materials and methods.

**FIGURE 7:**
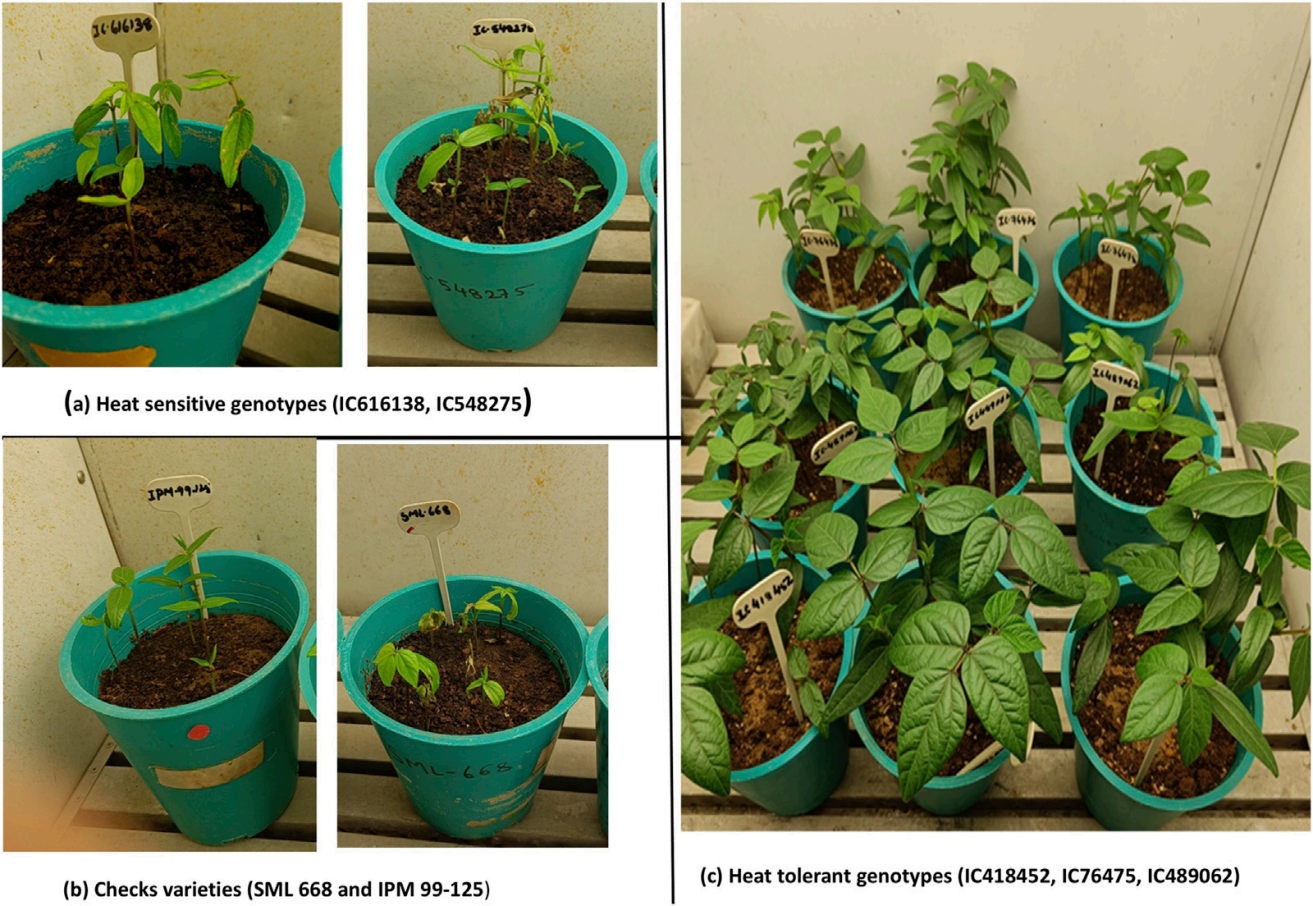
Growth chamber view of mungbean genotypes used for qRT-PCR. Under the heat stress treatment (SS) vigorous canopy growth was observed in heat stress tolerant genotypes *viz.*, IC76475, IC418452 and IC489062 **(C)**, while the check varieties which are popular for summer season-grown mungbean crop *viz.*, SML668 and IPM99-125 **(B)** showed poor growth and leaf burning at the elevated temperature of 40 °C. The heat-susceptible genotypes *viz.*, IC616138 and IC548275 **(A)** also showed poor growth and leaf burning.

**FIGURE 8:**
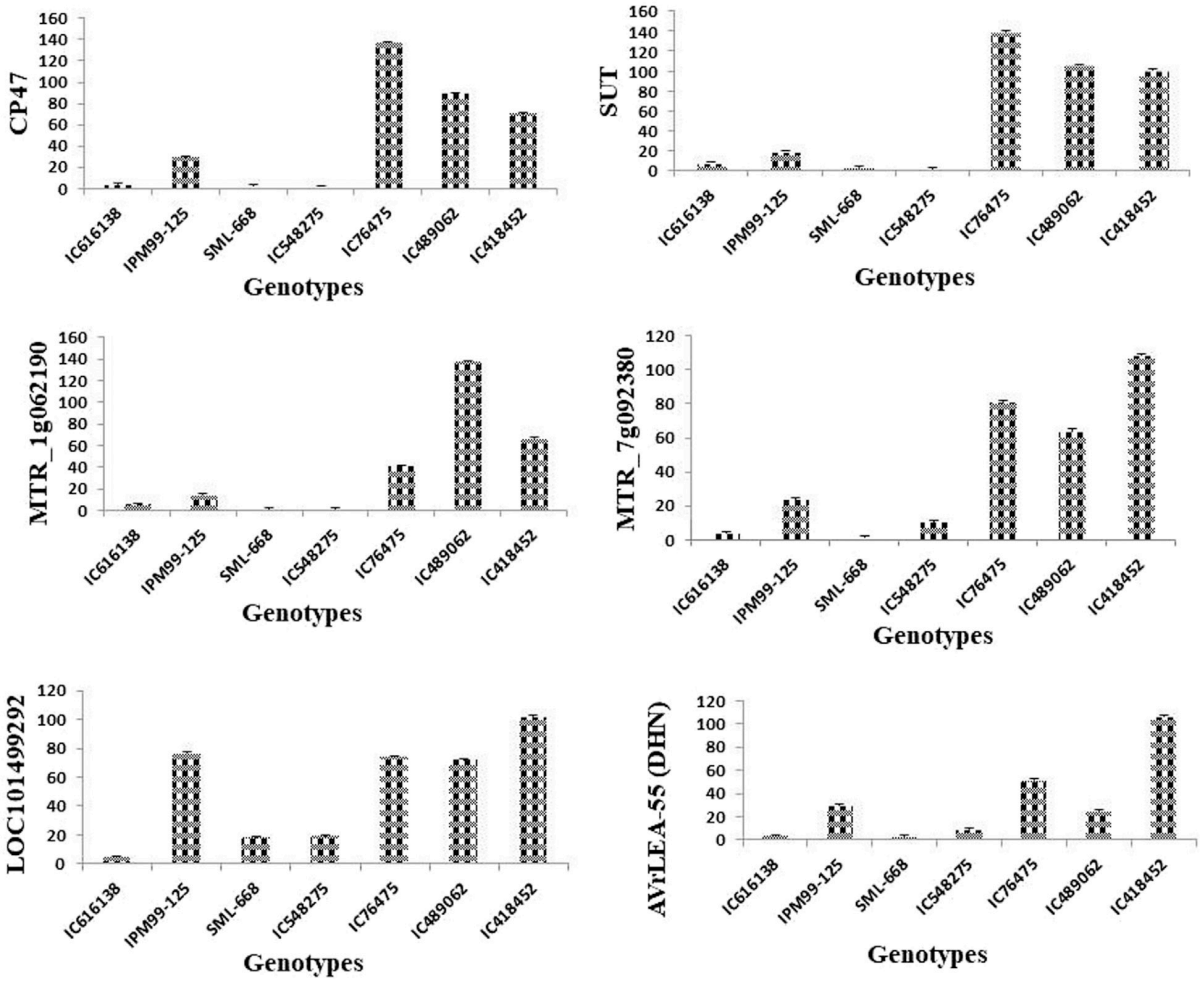
Effect of heat stress on the relative expression candidate genes [CP47, SUT, MTR_1g062190, MTR_7g092380, LOC101499292, VrLEA-55 (DHN)] involved in key physiological of heat stress tolerance. The analysis indicated a significantly higher level of expression for tolerant mungbean genotype (IC76475, IC418452, IC489062) in comparison to checks varieties (SML668, IPM99-125) and susceptible genotypes (IC616138, IC548275) used. The bars represent mean values of the relative normalized expression of genes ± SE.

The expression of all genes under study was significantly altered under heat-stress conditions (Figure 7). All these genes were found to be upregulated under heat stress conditions, however, the expression of IC76475 IC418452 and IC489062 mungbean genotype was relatively higher (Table 7; Figure 8). Conversely, the expression of the candidate genes in heat-sensitive mungbean genotypes *viz.*, IC616138 and IC548275 was downregulated as compared to control conditions.

**TABLE 7:** The expression statistics of key candidate genes involved in heat stress tolerance.

Gene name	Genotypes	*p*-value	p-adjV	Sig/non-sig	Up/downregulation
CP47	IC616138	0.02	0.03	S	up
	IPM99-125	0	0.01	S	up
	SML-668	0.16	0.18	NS	up
	IC548275	0.18	0.18	NS	up
	IC76475	0.04	0.05	S	up
	IC489062	0	0.01	S	up
	IC418452	0.02	0.03	S	up
SUT	IC616138	0.02	0.04	S	up
	IPM99-125	0.04	0.06	HS	up
	SML-668	0.14	0.17	NS	up
	IC548275	0.95	0.95	NS	up
	IC76475	0	0.01	S	up
	IC489062	0.01	0.02	S	up
	IC418452	0	0.01	S	up
MTR_1g062190	IC616138	0.12	0.17	NS	up
	IPM99-125	0.02	0.03	S	up
	SML-668	0.94	0.94	NS	up
	IC548275	0.18	0.21	NS	up
	IC76475	0.01	0.02	S	up
	IC489062	0.01	0.02	S	up
	IC418452	0.01	0.02	S	up
MTR_7g092380	IC616138	0.06	0.08	S	up
	IPM99-125	0.03	0.06	S	up
	SML-668	0.46	0.46	S	up
	IC548275	0.23	0.27	NS	up
	IC76475	0	0	S	up
	IC489062	0.01	0.03	S	up
	IC418452	0	0	S	up
LOC101499292	IC616138	0.01	0.01	S	up
	IPM99-125	0.02	0.03	S	up
	SML-668	0.04	0.04	S	up
	IC548275	0.12	0.12	NS	up
	IC76475	0	0.01	S	up
	IC489062	0	0.01	S	up
	IC418452	0	0.01	S	up
VrLEA-55 (DHN)	IC616138	0.05	0.06	S	up
	IPM99-125	0.01	0.02	S	up
	SML-668	0.06	0.07	S	up
	IC548275	0.3	0.3	NS	up
	IC76475	0.01	0.02	S	up
	IC489062	0	0	S	up
	IC418452	0	0	S	up

Abbreviation: p-adjV (P adjusted value), S (significant), NS (non-significant), HS (highly significant).

Expression profiling of key genes under heat stress revealed significant insights into the mungbean genotype’s responses. *CP47*, crucial for maintaining photosystem II (PSII) integrity, showed pronounced upregulation in all heat-tolerant genotypes (IC76475, IC418452, IC489062), indicating its role in maintaining photosynthesis under elevated atmospheric temperature. Conversely, heat-sensitive genotypes (IC616138, IC548275) exhibited negligible *CP47* expression under heat stress. Similarly, *SUT*, involved in sucrose transport displayed significant upregulation in all genotypes except IC548275 and SML-668, highlighting its role in energy supply and stress adaptation. *MTR_1g062190*, associated with general stress responses and membrane stabilization, exhibited high expression in all heat-tolerant genotypes, contrasting with minimal expression in heat-sensitive genotypes. *MTR_7g092380*, likely involved in protein folding under heat stress responses, showed a similar response. LOC101499292, important for glycosylation, displayed varying expression patterns among genotypes, being notably higher in IC489062, IC418452 and IC76475 compared to IC616138 and IC548275. *VrLEA-55* (DHN), known for its protective functions during abiotic stresses, also exhibited its role in enhancing stress tolerance.

## 4 Discussion

Rising atmospheric temperatures due to climate change are expected to have significant implications for global agricultural production, including that of mungbean. Mungbean is a warm-season legume, that thrives well between 28°C and 35°C, however, temperatures exceeding this range, particularly above 40°C, can lead to heat stress, causing flower and pod abortion, reduced seed filling and ultimately lower yields (Kaur et al., 2015). Globally over 43,000 mungbean collections are conserved, which can be used to identify superior genotypes for heat tolerance (Gayacharan et al., 2020). Therefore, in this study, we have screened diverse mungbean collections obtained from the Indian National Genebank. The mungbean germplasm included prominent mungbean varieties, local germplasm collections and landraces, collected from different parts of India (Table 1). We observed a good amount of variability available for heat stress tolerance traits in mungbean germplasm (Table 3). Moreover, many of the mungbean germplasm lines performed relatively better than the check varieties used in this study. The check varieties used in this study are recommended for their cultivation as summer crop in specific zones in India, for example, IPM 205-7 (Virat) for South and Central Zone, IPM 99-125 for Northeast Plain Zones, SML 668 for Punjab; and Pusa Vishal for Northwest Plain Zones (Tiwari and Shivhare, 2019). The inclusion of multiple check varieties in this experiment allowed for a robust comparison of trait expression under elevated heat stress conditions, providing a standard for identifying superior mungbean genotypes with relatively better heat stress tolerance.

The morphological variations and adaptive responses in mungbean were also reported earlier but with a very limited number of accessions, which are primarily elite lines of mungbean (Kaur et al., 2015; Sharma et al., 2016; Priya et al., 2023). Sharma and coworkers (2016) used 41 elite mungbean lines which included imported elite mungbean lines from The World Vegetable Centre, Taiwan for the evaluation against heat stress tolerance under pot conditions using morpho-physiological and biochemical traits, and identified five genotypes as heat tolerant genotypes. Kaur et al. (2015) studied reproductive biology using two mungbean cultivars (SML 832 and 668) in response to heat stress. Similarly, Priya and coworkers (2023) used two genotypes *viz.*, EC693369 as heat tolerant and KPS1 (EC592167) as heat sensitive to see the impact of heat stress on grain filling and other phenotypic characteristics. Patriyawaty et al. (2018) studied the genotypic responses to heat stress using five heat tolerant and five susceptible mungbean genotypes in a combination of physiological parameters and acclimation treatment. They observed a drastic reduction in shoot-root biomass and grain yield, but less drastic effects on acclimatization treatment. In a study, 17 mungbean genotypes were selected from a total of 116 genotypes based on the evaluation data for flowering, biomass, and yield attributes for further study on various parameters in response to heat stress (Basu et al., 2019). The study identified EC398889 as a heat tolerant mungbean genotype. The literature indicated that the previous studies have primarily focused on a narrow range of mungbean genotypes, mainly elite lines or released varieties, revealing a limited understanding of the genetic variability for heat stress tolerance, although a good number of mungbean *ex-situ* collections are conserved worldwide. However, our study used diverse mungbean collections and also revealed a good amount of variability for the traits studied (Tables 1, 3).

This study revealed varying responses of mungbean germplasm to heat stress. Each genotype showed a different magnitude of expression for each trait studied. The impact of heat stress is not limited to the traits studied but to overall phenotype. The heat stress alters each physiological and biochemical process thereby its phenotype (Kuppusamy et al., 2023). For example, Douglas et al. (2020) stated that, in summer season plants, there is a relative reduction within leaf area contrasted to the rainy season, also morphological alterations, namely, chlorosis, scorching and wilting have been noticed. The result of our study also revealed significant morpho-physiological changes in response to heat stress (Figure 2; Table 3). Most of the genotypes that performed better in normal sown conditions (RS) did not perform well during summer-sown (SS) conditions. Although some genotypes showed very good resilience towards elevated temperature their performance was better for multiple traits. Among such genotypes are IC418452, IC489062 and IC76475. There are other mungbean genotypes which were found superior for more than two traits *viz.*, IC39293, IC616215, IC398984, IC252010, IC398984, IC52078, IC76475 and IC623822 (Table 3).

The passport information of the heat-tolerant mungbean genotypes *viz.*, IC418452 (Andaman and Nicobar Islands), IC489062 (Rajasthan), and IC76475 (Delhi) indicates that they originate from geographically diverse regions of India, each representing distinct climatic zones (Table 1). This suggests that these genotypes have evolved independently under different environmental conditions, resulting in their diverse genetic backgrounds. Notably, they have developed heat stress tolerance, as the mungbean growing season in the Andaman and Nicobar Islands coincides with the hot summer months, while in Rajasthan, the kharif season often experiences temperatures reaching up to 40°C. The variation in trait expression, as detailed in Table 3 and confirmed by qRT-PCR analysis (Figure 8), further emphasizes their genetic diversity. Genotype IC418452 demonstrates superior performance across seven traits, including the number of pods per plant, number of pods per cluster, number of seeds per pod, membrane stability index, chlorophyll content, pollen viability, and 100-seed weight. Similarly, IC76475 excels in traits such as the number of pods per cluster, number of seeds per pod, and chlorophyll content. Although IC489062 shows superiority in five traits, its performance varies compared to the other genotypes, highlighting the unique strengths of each. Here, these results gives an opportunity for more detailed genetic analysis to identify genes, QTLs and other regulatory factors involved in imparting heat stress tolerance in these genotypes. Further, to develop robust heat stress tolerance in mungbean, these genotypes can serve as the donors for introgression breeding.

In the SS the maximum number of seed per pod was found in genotypes *viz.*, IC76475, IC418452, IC507517 and IC76585. Similarly, promising genotypes for other traits were also identified (Table 3). Genotypes with better chlorophyll contents are considered as promising as chlorophyll is directly related to the photosynthetic capacity of the plant, a reduction in chlorophyll content due to heat stress leads to reduced plant yield (Roy et al., 2021). Although our results indicated the reduced chlorophyll content in heat stress conditions, some genotypes showed higher chlorophyll content (Table 3). Such genotypes can be useful for further in-depth study. Heat stress also adversely affects the leaf by reducing its water potential and turgor pressure which leads to reduced leaf area and yield performance (Hasanuzzaman et al., 2013). The reduction in leaf area is also an adaptive response of plants to retain more soil moisture. Conversely, a larger leaf area is associated with lower leaf temperature, higher CTD and also higher grain yield. Although in mungbean such studies are not available, similar correlation patterns are observed in this study (Figure 4). In general, the correlation statistics among variables under both the conditions *viz.*, SS and RS indicate that the expression of most of the traits studied is independent, except for a few (Figure 4). The traits CT and CTD showed strong and negative correlation and while traits related to leaf dimension showed a positive correlation (Figure 4). Canopy temperature and canopy temperature depression (CTD) play a great role in the adaptation of plants under elevated temperature regimes. It is reported that higher CTD is associated with heat stress resilience and crop yield (Lepekhov, 2022). The result of Huang et al. (2021) stated that heat stress causes great disruption in the photosynthesis apparatus, therefore higher CTD efficiency of a plant is very crucial for its yield under heat stress conditions.

Huang et al. (2021) stated that a higher MSI indicates better membrane stability, meaning the cell membranes remain more intact and functional under stress conditions. Conversely, a lower MSI suggests higher membrane damage and instability, indicating greater susceptibility to stress. Our study revealed that maximum membrane damage was noticed in RMG-344, IC121301 and IC488554 and minimum damage was seen in genotypes IC148530, IC418452, IC76475 and EC251557 in the summer season showing high tolerance for high-temperature stress in compared to RS. The pollen grains became less viable and less vigorous in heat-stressed plants (Snider and Oosterhuis, 2012). Under summer-sown (SS) conditions, the maximum pollen viability was observed in lines IC418452, IC39428, IC314609 and IC313547.

The heat stress susceptibility index (HSI) is a measure of a genotype’s sensitivity to heat stress, with higher values indicating greater susceptibility. The HSI is considered as a good criterion for the identification of genotypes for heat stress tolerance (Lamba et al., 2023). The HSI ranged from 1.23 to −0.1. The genotypes IC623822 (0.20), IC616201 (0.16) and IC489062 (−0.10) exhibited lower HSI than the best check variety SML-668 (0.32). The genotype IC489062 performed best among others, which is also one of the three best genotypes that were found superior for multiple traits. The HSI of the other two genotypes *viz.*, IC418452 (0.92) and IC76475 (0.67) was of moderate level, which indicates that these genotypes may not be good yielders.

The principal component analysis (PCA) revealed the uniform distribution of diversity among genotypes indicating that the genotypes are diverse and have unique characteristics. The first five PCs could reveal only approximately 72% of the entire variation present in the genotypes under both the conditions, i.e., RS and SS. Although the level of diversity explained under both conditions is the same, deviations among variables and genotypes are observed in their 2-D space (Figure 5) indicating the effect of heat stress on the expression of genotypes. The relationship among the morpho-physiological parameters in PCA showed a similar pattern to correlation results. The Agglomerative Hierarchical Clustering (AHC) method also showed shifts in the clustering pattern of genotypes due to heat stress. Notably, genotypes IC76475, IC418452 and IC489062, which were identified as heat stress-tolerant for multiple traits, clustered together under SS conditions (Figure 6). This suggests that AHC can be an effective tool for the easy identification and visualization of genotypes with similar characteristics.

This study identified some very promising mungbean genotypes for heat stress tolerance, which were further validated in artificial conditions as discussed in materials and methods. The genotypes IC418452, IC489062 and IC76475 showed significant upregulation of key candidate genes in qRT-PCR analysis indicating their inherent heat tolerance efficiency. Moreover, these genotypes also outperformed the best check varieties used in this study for the traits studied, this signifies the importance of the outcomes from this study. Overall, this study yields some very important findings such as the impact of heat stress on key morpho-physiological characteristics the relationship among traits, and trait-specific genotypes which can be utilized as donors in mungbean breeding programs for heat stress resilience.

## 5 Conclusion

This study provides a comprehensive analysis of the effects of heat stress on various physiological, and morphological traits in 87 mungbean germplasm collections. This study revealed significant insights into the complex interactions and adaptive responses of mungbean under elevated temperatures. Heat stress significantly affects morphological as well as physiological parameters. The findings of this study were further validated using important key candidate genes through qRT-PCR analysis. While previous studies have explored similar aspects, they have used a very limited number of genotypes and primarily elite lines or released varieties. This suggests a research gap in understanding the genetic variability available in mungbean germplasm, thereby limiting the potential uses of the findings. Hence, this study will supplement the understanding in this area of research and also give impetus to the mungbean breeding programs to develop heat stress resilient mungbean cultivars. The identified heat-tolerant mungbean germplasm lines such as IC418452, IC489062, and IC76475 will have significant implications for mungbean improvement, particularly in the context of the developing heat stress resilience in the crop amid climate change. However, heat stress tolerance is a complex trait influenced by multiple genetic and environmental factors, presenting ongoing challenges in developing heat-tolerant mungbean cultivars. The integration of high-throughput precision phenotyping in controlled environments, combined with genomics and metabolomics approaches can provide deeper insights into the physiological mechanisms and genetic factors of heat stress tolerance, thereby accelerating breeding efforts.

## Data Availability

The raw data supporting the conclusions of this article will be made available by the authors, without undue reservation.
